# ISL1 regulates lung branching morphogenesis *via* Shh signaling pathway

**DOI:** 10.1016/j.jbc.2023.105034

**Published:** 2023-07-11

**Authors:** Ruiqi Huang, Chujing Zhang, Yuting Zheng, Wei Zhang, Huarong Huang, Mengsheng Qiu, Jianying Li, Feixue Li

**Affiliations:** Zhejiang Key Laboratory of Organ Development and Regeneration, Institute of Developmental and Regenerative Biology, College of Life and Environmental Sciences, Hangzhou Normal University, Hangzhou, People’s Republic of China

**Keywords:** Isl1, Shh, lung branching, signal pathway, transcription factor

## Abstract

Lung branching morphogenesis relies on a complex coordination of multiple signaling pathways and transcription factors. Here, we found that ablation of the LIM homeodomain transcription factor Islet1 (*Isl1*) in lung epithelium resulted in defective branching morphogenesis and incomplete formation of five lobes. A reduction in mesenchymal cell proliferation was observed in *Isl1*^*ShhCre*^ lungs. There was no difference in apoptosis between the wild-type (*Shh*^*Cre*^) and *Isl1*^*ShhCre*^ embryos. RNA-Seq and *in situ* hybridization analysis showed that *Shh*, *Ptch1*, *Sox9*, *Irx1*, *Irx2*, *Tbx2*, and *Tbx3* were downregulated in the lungs of *Isl1*^*ShhCre*^ embryos. ChIP assay implied the *Shh* gene served as a direct target of ISL1, since the transcription factor ISL1 could bind to the *Shh* epithelial enhancer sequence (MACS1). Also, activation of the Hedgehog pathway *via* ectopic gene expression rescued the defects caused by *Isl1* ablation, confirming the genetic integration of Hedgehog signaling. In conclusion, our works suggest that epithelial *Isl1* regulates lung branching morphogenesis through administrating the Shh signaling mediated epithelial-mesenchymal communications.

As a respiratory organ, the main function of the lung is to realize the effective gas exchange between the blood and the external environment to maintain life activities. Lung development undergoes a series of developmental events, including branching morphogenesis and alveolar differentiation (1, 2). Airways develop sequentially through early epithelial duct branching and late air sac separation. Lung branching is a highly coordinated process that generates a complex network of gas exchange units. In mice, branching morphogenesis begins with primary lung bud proliferation and continues to grow into the surrounding mesenchyme at E10.5 (3). There is an intimate crosstalk between the epithelium and the mesenchyme during branching (4, 5, 6). Lung branching relies on a complex coordination of multiple signaling pathways and transcription factors, but the underlying precise mechanisms that control lung branching remain elusive (7, 8, 9, 10).

*Shh* is expressed in the epithelial cells at the onset of lung growth at day E9.5. Deletion of *Shh* results in failure of branching and growth after the formation of the primary lung buds (11). The proliferation of mesenchymal cells is significantly decreased in the *Shh* knockout mice (11, 12, 13). These results highlight the importance of *Shh* in regulating lung branching. Genetic rescue experiments revealed that transcription factors *Tbx2* and *Tbx3* act mainly downstream of epithelial Shh signaling to promote mesenchymal proliferation and maintain branching morphogenesis (14). *Sox9* also plays an important role in lung branching, and ablation of *Sox9* in epithelial cells results in dramatic defects in lung branching (15). *Sox9* promotes proper branching morphogenesis by controlling the balance between proliferation and differentiation. Iroquois homeobox (Irx) genes are reported to be involved in the regulation of proximal-distal patterning during lung development (16, 17). The expression of *Irx1*-*3* and *Irx5* is restricted to branching lung epithelium, and knockdown of all lung *Irx* genes together significantly reduces distal branching events and increases proximal tubule dilation *in vitro*.

Transcription factor *Isl1* has been reported to play a critical role in organ patterning (18, 19, 20, 21). *Isl1* has been reported to regulate tracheo-esophageal separation and lung lobation (22). The exact molecular mechanism of *Isl1* during lung branching remains to be determined. Here, we found that ablation of *Isl1* in the lung epithelium resulted in defects in branching morphogenesis. *Shh* was identified as a target gene of ISL1 in the lung epithelium. Activation of Hedgehog signaling by Purmorphamine treatment or by ectopic *Ihh* expression would rescue the defects triggered by Isl1 deletion. Thus, we revealed that Isl1 regulates lung branching morphogenesis through Shh signaling.

## Results

### Loss of Isl1 results in impaired lung branching morphogenesis

**Figure 1:**
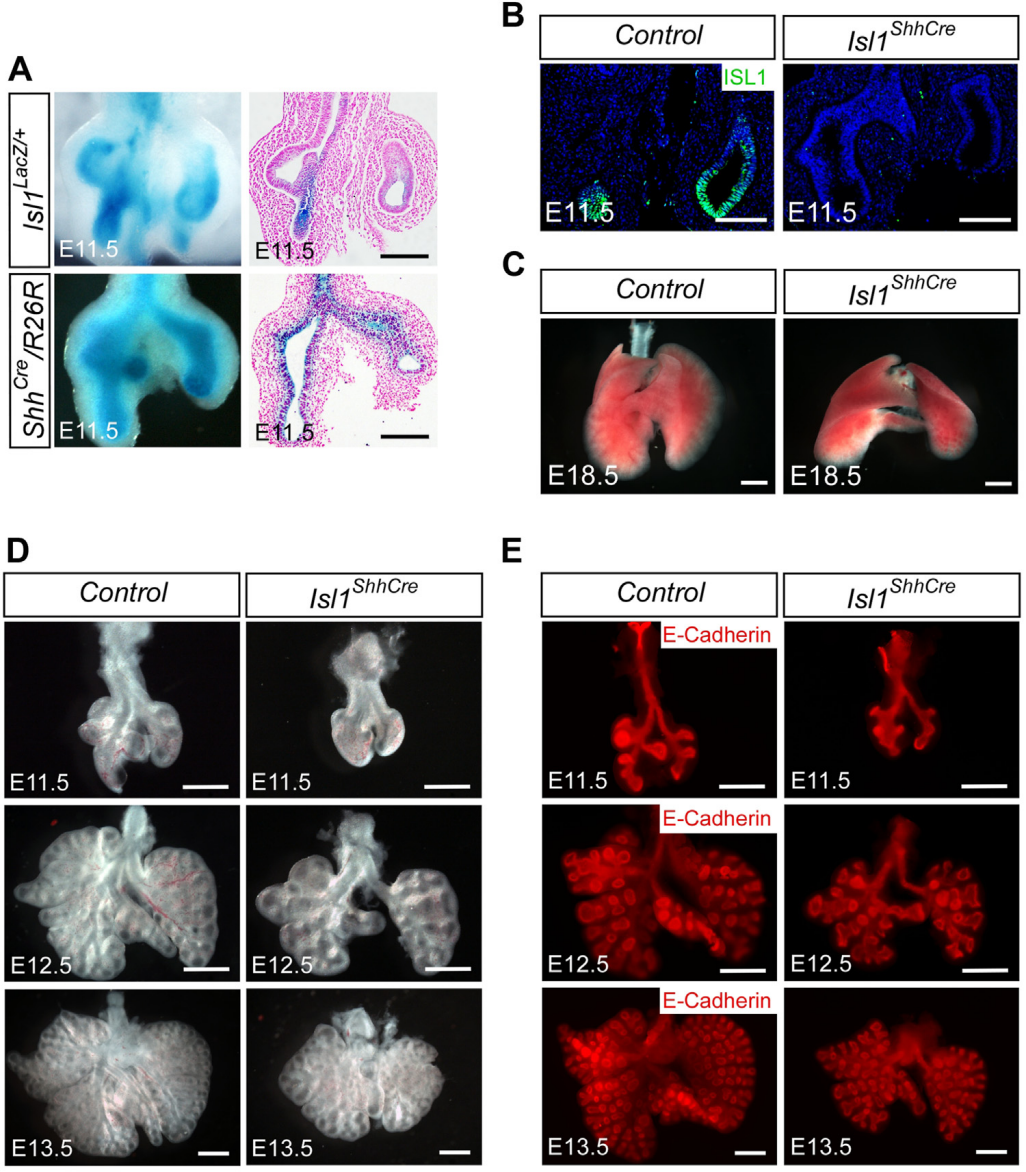
***Isl1* is required for mouse lung branching morphogenesis.***A*, X-Gal staining showing *Isl1* expression in the lung of *Isl1*^*LacZ*^ knock-in mice at E11.5. X-Gal staining showing Shh-Cre activity in *Shh*^*Cre*^/*R26R* mice at E11.5. *B*, immunofluorescence staining with ISL1 antibody on the sections of E11.5 control (*Shh*^*Cre*^) and *Isl1*^*ShhCre*^ lungs. *C*, morphology of lungs of control (*Shh*^*Cre*^) and *Isl1*^*ShhCre*^ mice at E18.5. *D*, morphology of E11.5, E12.5, and E13.5 control (*Shh*^*Cre*^) and *Isl1*^*ShhCre*^ lungs. *E*, immunofluorescence staining with E-cadherin antibody of E11.5, E12.5, and E13.5 control (*Shh*^*Cre*^) and *Isl1*^*ShhCre*^ lungs. Scale bars, 200 μm (*A* and *B*), 1 mm (*C*), 500 μm (*D* and *E*).

The expression of *Isl1* in the lung was determined using *Isl1*^*LacZ*^ knock-in allele. *Isl1* expression was detected in the lung epithelium as early as E11.5 (Fig. 1*A*), suggesting a role in lung development. To investigate the potential function of *Isl1* in the development of lung, we generated mice with epithelial cell specific deletion of *Isl1* by crossing *Shh*^*Cre*^ with *Isl1*^*fl*^ mice (*Isl1*^*ShhCre*^). The fusion of all four right lung lobes was observed in *Isl1*^*ShhCre*^ embryos (100% penetration) (Fig. 1*C*). Immunofluorescence and morphological analyses demonstrated the deletion of ISL1 (Fig. 1*B*) and the defect of lung branching process in *Isl1*^*ShhCre*^ embryos (Fig. 1, *D* and *E*). The results showed that lung branching morphogenesis was delayed in the *Isl1*^*ShhCre*^ mutant embryos from E11.5 to E13.5. These data suggest that *Isl1* is required for lung branching morphogenesis.

### Isl1 deletion reduces the proliferation of mesenchymal cells without disturbing the apoptosis

**Figure 2:**
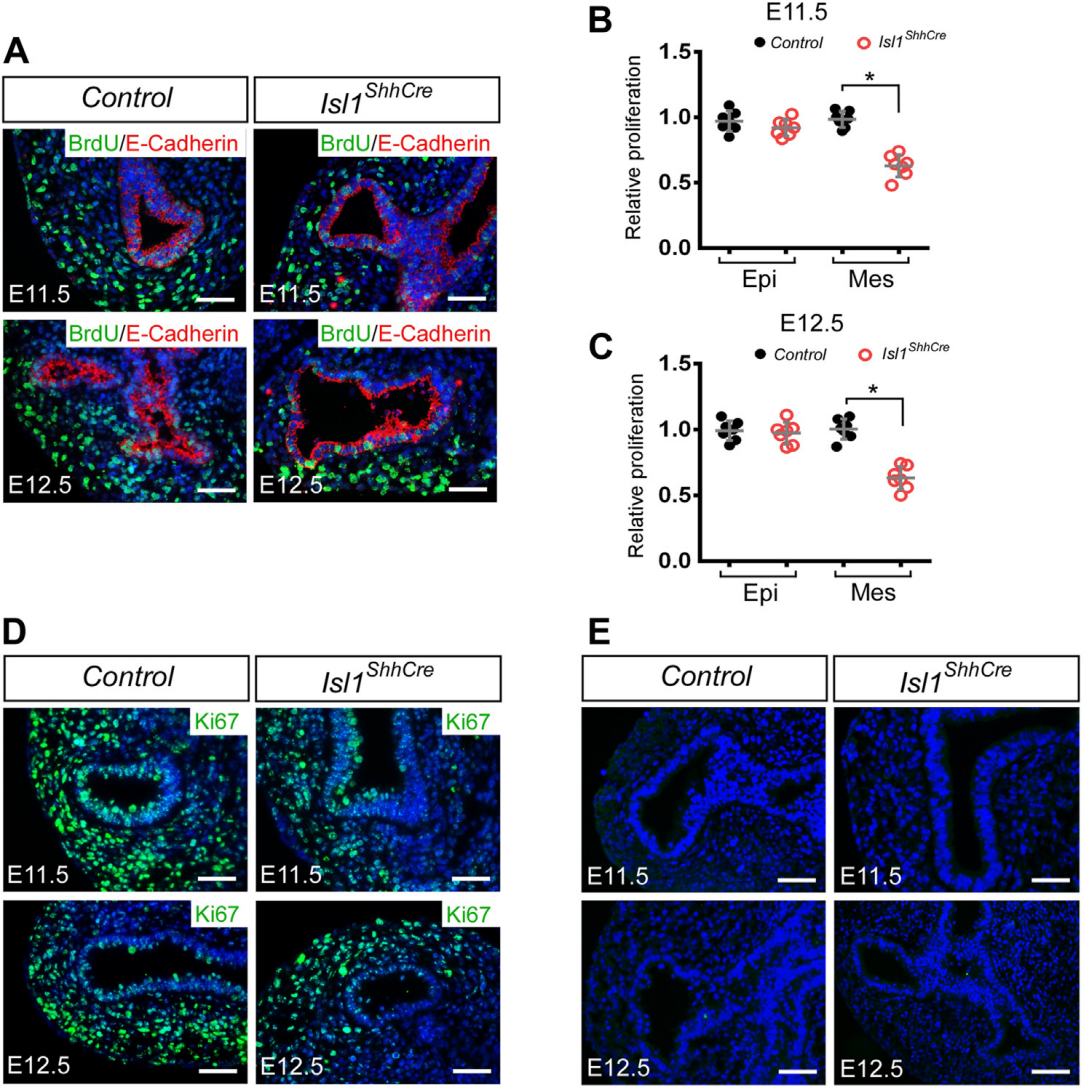
**Deletion of *Isl1* in the lung epithelial cells reduces of cell proliferation.***A*, double immunofluorescence staining with E-cadherin and BrdU on the sections of E11.5 and E12.5 control (*Shh*^*Cre*^) and *Isl1*^*ShhCre*^ lungs. *B*, quantification of the relative epithelial and mesenchymal proliferation determined by the BrdU incorporation assay at E11.5. *C*, quantification of the relative epithelial and mesenchymal proliferation determined by the BrdU incorporation assay at E12.5. Error bars represent standard deviations (n = 7 samples). The proliferation rate of control (*Shh*^*Cre*^) was normalized to 1. *D*, immunofluorescence staining with Ki67 on the sections of E11.5 and E12.5 control (*Shh*^*Cre*^) and *Isl1*^*ShhCre*^ lungs. *E*, TUNEL staining on the sections of E11.5 and E12.5 lungs in *Shh*^*Cre*^ (Control) and *Isl1*^*ShhCre*^ mice. ∗, *p* < 0.05. Scale bars, 100 μm.

To investigate the cellular mechanisms responsible for defective lung branching morphogenesis in *Isl1* mutants, we examined cell proliferation and apoptosis. E-cadherin has been characterized and used as an early-airway epithelial marker (23). BrdU was counterstained with E-cadherin to determine the proliferation of epithelial cells in the lung. The results of immunofluorescence showed that BrdU-labeled mesenchymal cells were significantly reduced in *Isl1*^*ShhCre*^ lungs from E11.5 to E12.5 compared with wild type (Fig. 2, *A*–*C*). Anti-Ki67 antibody (proliferation marker) was also used to detect cell proliferation, which also showed a reduction in mesenchymal cell proliferation through all detected stages (Fig. 2*D*). To determine whether defects in lung branch morphogenesis were due to increased abnormal cell apoptosis, we performed TUNEL assays on tissue sections from E11.5 to E12.5. There was no obvious difference in apoptosis between the control (*Shh*^*Cre*^) and *Isl1*^*ShhCre*^ embryos (Fig. 2*E*). The results suggest that *Isl1* is critical for cell proliferation during lunging branch morphogenesis but not for cell survival. Decreased mesenchymal cell proliferation may account for the developmental defect of the lung branching in *Isl1*^*ShhCre*^ embryos.

### Loss of Isl1 results in differential expression of genes critical for branching morphogenesis

**Figure 3:**
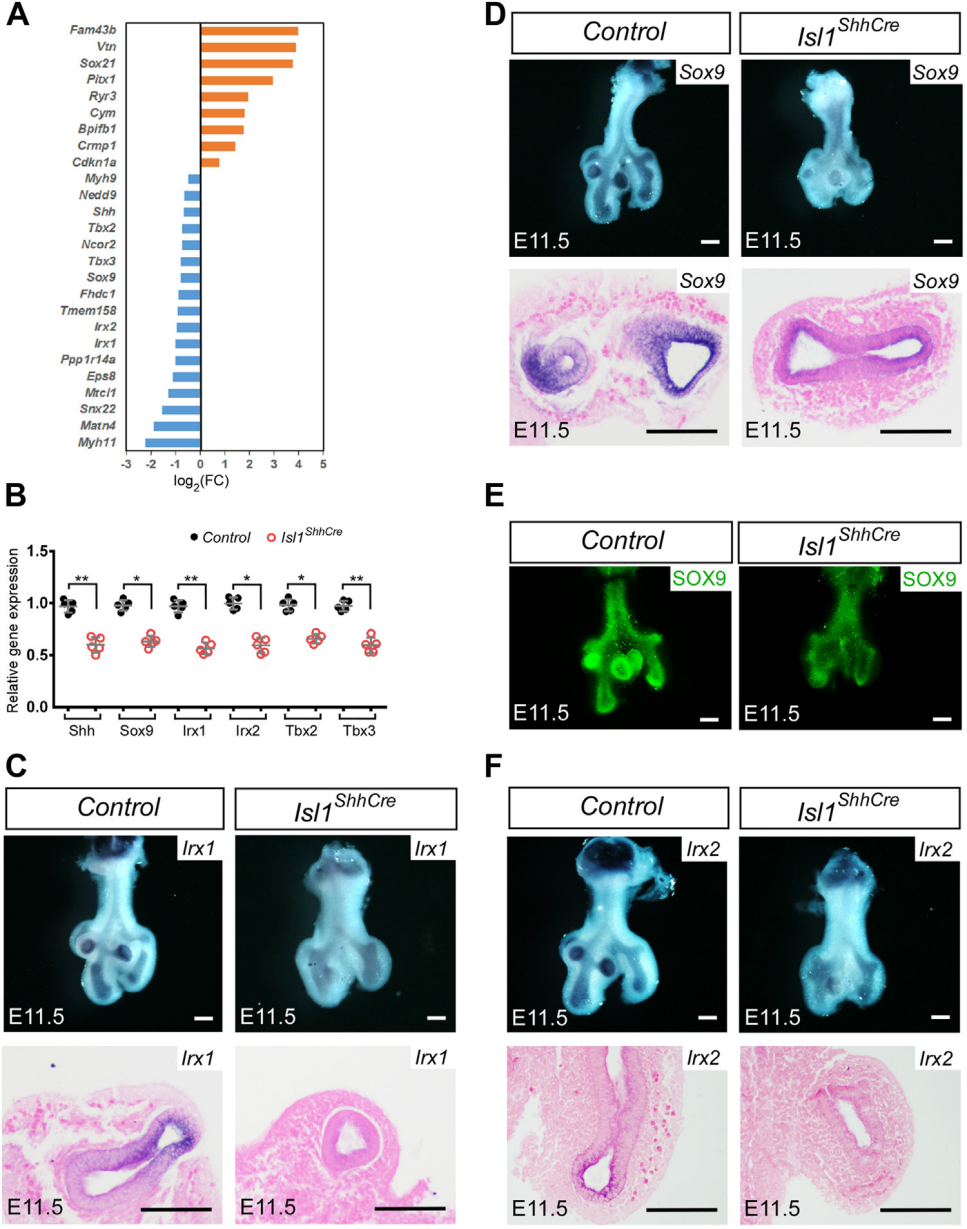
**Changes in gene expression in the lungs of the *Isl1***^***ShhCre***^**mutant.***A*, representative examples of differentially expressed genes in the *Isl1*^*ShhCre*^ as revealed by RNA-Seq analysis (≥1.5-fold, <5% false discovery rate). *B*, quantitative RT-PCR demonstrates the transcription of *Shh*, *Sox9*, *Irx1*, *Irx2*, *Tbx2*, and *Tbx3* in E11.5 control (*Shh*^*Cre*^) and *Isl1*^*ShhCre*^ lungs. The expression of genes in control (*Shh*^*Cre*^) was normalized to 1. Error bars represent standard deviations (n = 5 samples). *C*, whole-mount and section *in situ* hybridization showing the expression of *Irx1* in the *Shh*^*Cre*^ (Control) and the *Isl1*^*ShhCre*^ lungs at E11.5. *D*, whole-mount and section *in situ* hybridization showing the expression of *Sox9* in the *Shh*^*Cre*^ (Control) and the *Isl1*^*ShhCre*^ lungs at E11.5. *E*, whole-mount immunofluorescence staining with SOX9 of E11.5 control (*Shh*^*Cre*^) and *Isl1*^*ShhCre*^ lungs. *F*, whole-mount and section *in situ* hybridization showing the expression of *Irx2* in the *Shh*^*Cre*^ (Control) and the *Isl1*^*ShhCre*^ lungs at E11.5. ∗, *p* < 0.05. ∗∗, *p* < 0.01. Scale bars, 200 μm.

To explore the molecular mechanism by which *Isl1* deletion caused lung branching defects, gene expression profiles were analyzed by RNA-Seq using RNA extracted from E11.5 lung tissues from mutant (*Isl1*^*ShhCre*^) and wild-type control (*Shh*^*Cre*^) mice to identify the potential downstream target genes of *Isl1*. We uncovered dozens of protein-coding genes that were differentially expressed (≥1.5-fold, <5% false discovery rate) in the *Isl1*^*ShhCre*^ mutants *versus* the controls (Fig. 3*A*). Among them, the expression of multiple genes known to be critical for lung branching changed significantly. Quantitative real-time PCR using RNAs purified from independent samples confirmed the altered expression of *Shh*, *Sox9*, *Irx1*, *Irx2*, *Tbx2*, and *Tbx3* (Fig. 3*B*). *In situ* hybridization and immunofluorescence also confirmed the downregulation of *Sox9*, *Irx1*, and *Irx2* (Fig. 3, *C*–*F*). These results indicate that the expression of all these genes is dependent on ISL1 in the lung epithelium.

### Shh gene is regulated by Isl1 in lung epithelium

**Figure 4:**
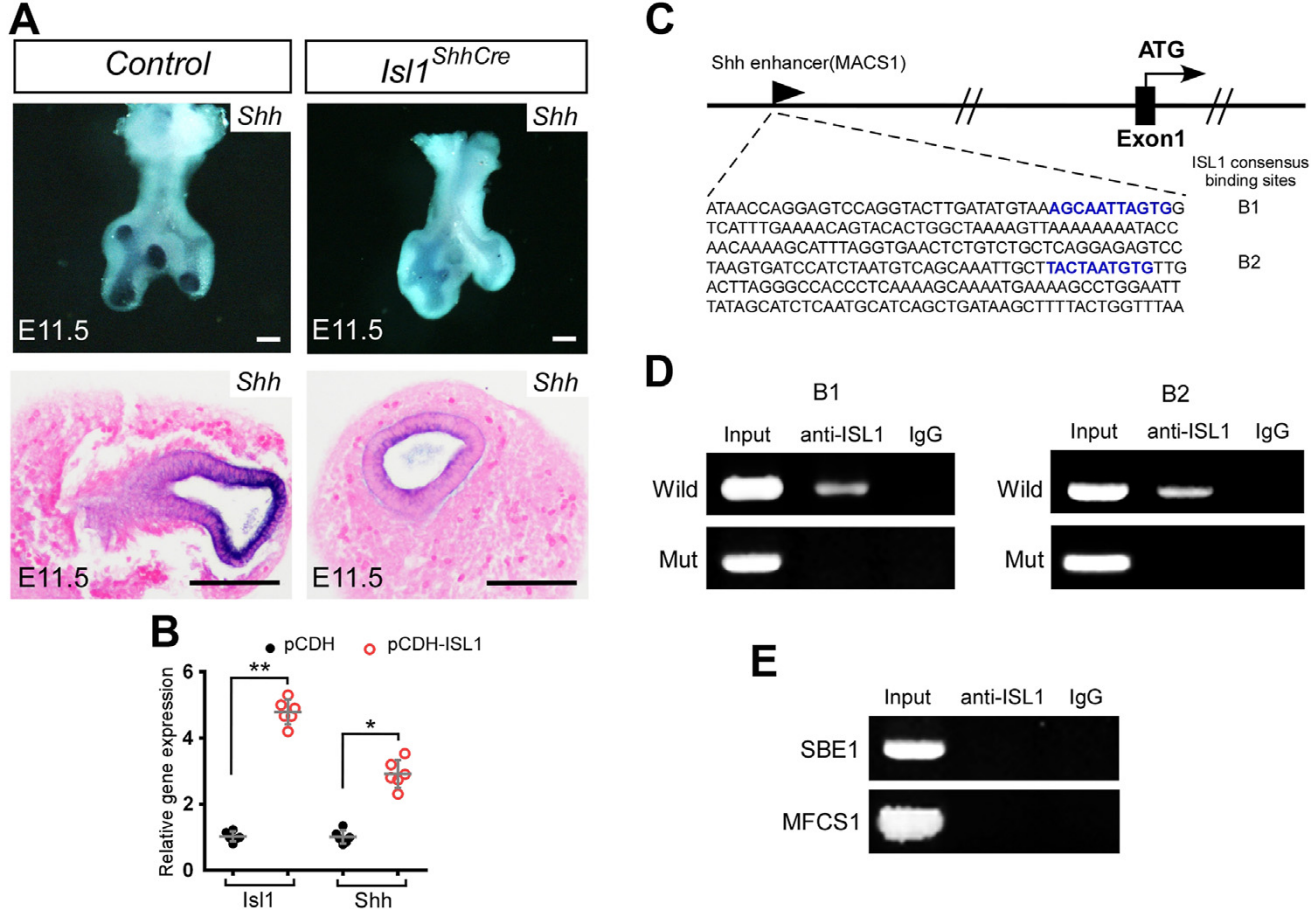
***Isl1* sustains the Hedgehog pathway *via* regulation of the *Shh* expression.***A*, whole-mount and section *in situ* hybridization showing the expression of *Shh* in the *Shh*^*Cre*^ (Control) and the *Isl1*^*ShhCre*^ (Mutant) lungs at E11.5. *B*, quantitative RT-PCR demonstrates the transcription of *Isl1* and *Shh* in primary lung cells cultures. The expression of genes in control (pCDH) was normalized to 1. Error bars represent standard deviations (n = 6 samples). *C*, schematic representation of the *Shh* epithelial enhancer sequence (MACS1). The consensus binding sites for ISL1 were highlighted (B1, B2). *D*, ChIP assays showed the binding of ISL1 on B1 and B2. *E*, ChIP assays showed no binding of ISL1 on *Shh* brain enhancer SBE1 or *Shh* limb bud-specific enhancer MFCS1 in wild type lungs. ∗, *p* < 0.05. ∗∗, *p* < 0.01. Scale bars, 200 μm.

During branching morphogenesis, there is an intimate crosstalk between the epithelium and the mesenchyme. We hypothesized that secreted signaling molecule SHH might be the downstream target of Isl1 in the epithelium through which Isl1 controls the cellular activities of the mesenchyme. The results of *in situ* hybridization confirmed the downregulation of *Shh* in the *Isl1*^*ShhCre*^ mutant (Fig. 4*A*). Overexpression of *Isl1* could significantly induce *Shh* expression in primary lung cells (Fig. 4*B*). It is known that the expression of *Shh* in the lung epithelium is regulated by the MACS1 (chr5:29416996-29417802) sequence. Two ISL1 consensus binding sites were found in the MACS1 sequence (Fig. 4*C*). The ChIP analysis showed the binding of ISL1 to the MACS1 sequence of the *Shh* gene (Fig. 4*D*). In addition, the ChIP analysis showed no binding of ISL1 to the *Shh* brain enhancer SBE1 (Chr5: 26858065- 26858607) or *Shh* limb bud-specific enhancer MFCS1 (chr5: 29520611-29519698) (Fig. 4*E*). Our data demonstrate that *Shh* mediates the effects of *Isl1* on lung branching development.

### Tbx2 and Tbx3 are upregulated by hedgehog signaling activation

**Figure 5:**
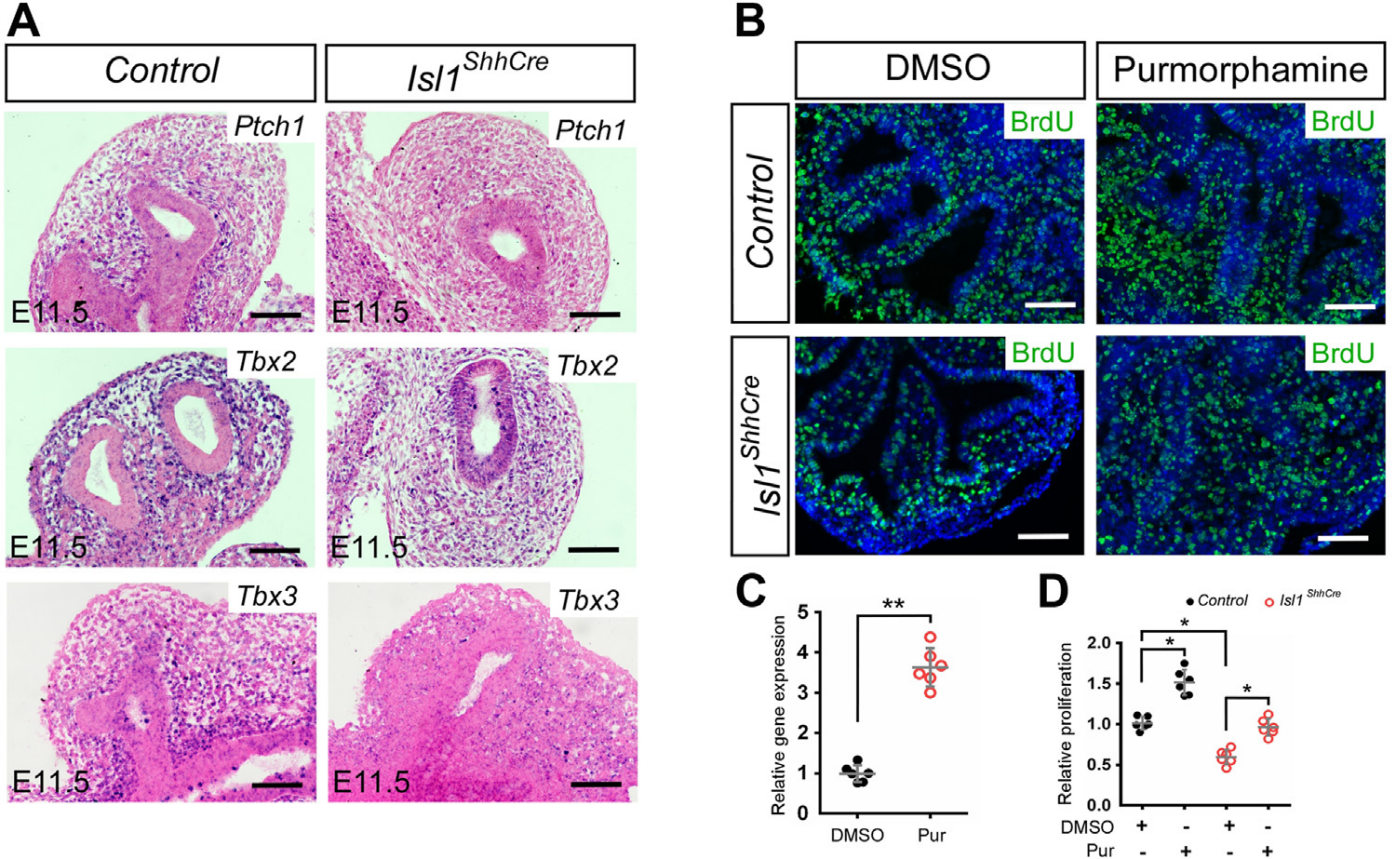
**Deletion of *Isl1* results in the downregulation of *Ptch1*, *Tbx2*, and *Tbx3*.***A*, *in situ* hybridization showing the expression of *Ptch1*, *Tbx2*, and *Tbx3* in the *Shh*^*Cre*^ (Control) and the *Isl1*^*ShhCre*^ lungs at E11.5. *B*, BrdU labeling analysis were performed on sections of organ cultures. *C*, quantification of the relative *Ptch1* expression after Purmorphamine (Pur) treatment. The expression of *Ptch1* in control (DMSO) was normalized to 1. Error bars represent standard deviations (n = 6 samples). *D*, quantification of the relative mesenchymal proliferation determined by the BrdU incorporation assay. The proliferation rate of control (DMSO) was normalized to 1. Error bars represent standard deviations (n = 6 samples). ∗, *p* < 0.05. ∗∗, *p* < 0.01. Scale bars, 100 μm.

*In situ* hybridization was used to detect the activity of Hedgehog signaling in the mesenchyme of *Isl1*^*ShhCre*^ mutant lungs. The expression of *Ptch1*, a marker gene for active Hedgehog signaling, is reduced in the mesenchyme of *Isl1*^*ShhCre*^ mutant lungs (Fig. 5*A*). The results showed that the activity of Hedgehog signaling in the mesenchyme of *Isl1*^*ShhCre*^ mutant was reduced. Expression of *Tbx2* and *Tbx3* was also decreased in the mesenchyme of *Isl1*^*ShhCre*^ mutant lungs (Fig. 5*A*). The results are consistent with reports that expression of *Tbx2* and *Tbx3* in the mesenchyme depends on epithelial-derived Shh signaling. Tbx2 and Tbx3 have been reported to maintain lung mesenchymal proliferation. Organ culture experiments were used to verify whether activation of Hedgehog signaling could rescue the cell proliferation defects in *Isl1*^*ShhCre*^ mutant lungs. The results indicated that mesenchymal cell proliferation was increased significantly after Purmorphamine treatment (Fig. 5, *B*–*D*). The increase of *Ptch1* expression indicated that Hedgehog signaling is activated after Purmorphamine treatment (Fig. 5*C*).

### Activation of Hedgehog signaling rescues the defects of lung branching *in vivo*

**Figure 6:**
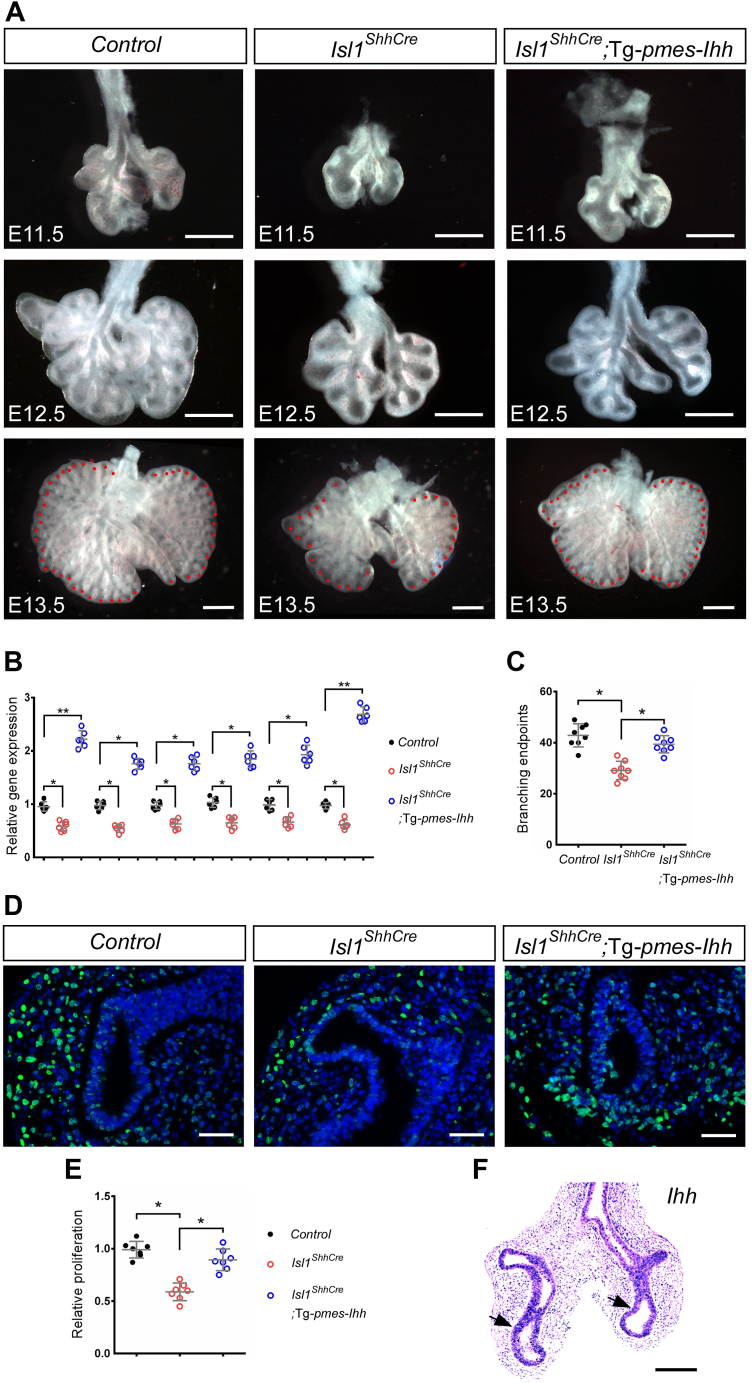
**Reactivation of Hedgehog signaling rescues lung development in *Isl1***^***ShhCre***^**mutant embryos.***A*, morphological analysis of E11.5 - E13.5 *Shh*^*Cre*^ (Control), *Isl1*^*ShhCre*^ and *Isl1*^*ShhCre*^; Tg-*pmes*-*Ihh* lungs. *B*, quantitative RT-PCR demonstrates the transcription of *Ptch1*, *Sox9*, *Irx1*, *Irx2*, *Tbx2*, and *Tbx3* in E13.5 *Shh*^*Cre*^ (Control), *Isl1*^*ShhCre*^, and *Isl1*^*ShhCre*^; Tg-*pmes*-*Ihh* lungs. Error bars represent standard deviations (n = 6 samples). *C*, distal epithelial tips of lungs were indicated by red dots in (*A*). The number of distal epithelial tips was counted. Error bars represent standard deviations (n = 8 samples). *D*, BrdU labeling analysis were performed on sections of E11.5 *Shh*^*Cre*^ (Control), *Isl1*^*ShhCre*^, and *Isl1*^*ShhCre*^; Tg-*pmes*-*Ihh* lungs. *E*, quantification of the relative mesenchymal proliferation determined by the BrdU incorporation assay. Error bars represent standard deviations (n = 7 samples). The proliferation rate of control (*Shh*^*Cre*^) was normalized to 1. *F*, section *in situ* hybridization showing the epithelial expression of *Ihh* in the *Isl1*^*ShhCre*^; Tg-*pmes*-*Ihh* lungs at E11.5. ∗, *p* < 0.05. ∗∗, *p* < 0.01. Scale bars: 500 μm (*A*), 100 μm (*D*), 200 μm (*F*).

We conditionally overexpressed a Hedgehog ligand *Ihh* in the lung epithelium using a transgenic allele (Tg-*pmes*-*Ihh*). Both *Ihh* and *Shh* could activate the same cell signaling pathway (24). The defect of lung branching in *Isl1*^*ShhCre*^ embryos was rescued by overexpression of *Ihh* in *Isl1*^*ShhCre*^; Tg-*pmes*-*Ihh* embryos (Fig. 6*A*). Previous reports have demonstrated that *Sox9*, *Irx1*, *Irx2*, *Tbx2*, and *Tbx3* are the downstream target genes of Hedgehog signaling pathway (14, 25, 26). The upregulation of *Ptch1*, *Sox9*, *Irx1*, *Irx2*, *Tbx2*, and *Tbx3* expression was detected in *Isl1*^*ShhCre*^; Tg-*pmes*-*Ihh* (Fig. 6*B*). The number of lung branching ends significantly increased in *Isl1*^*ShhCre*^; Tg-*pmes*-*Ihh* compared to that in the *Isl1*^*ShhCre*^ embryos (Fig. 6*C*). Overexpression of *Ihh* rescued the lung developmental defects due to *Isl1* ablation, confirming the genetic integration of Hedgehog signaling.

## Discussion

Lung branching morphogenesis is a complex process that generates a tree-like network consisting of proximal conducting airways and distal alveoli (1, 27). In this study, we observed an obvious branching defect as early as E11.5 in the *Isl1*^*ShhCre*^ mutant embryos, indicating an important function of *Isl1* at the beginning of branching. Shh signaling activity was downregulated in the *Isl1*^*ShhCre*^ mutant embryos. And activation of Hedgehog signaling could rescue the defect of lung branching.

LIM homeodomain transcription factor *Isl1* serves as a marker for patterning and cell type specification in many developmental processes (20, 28, 29, 30). *Isl1* highly expresses in lung epithelial cells as early as day E11.5, suggesting that it plays an important role in early lung development. Deletion of *Isl1* in the lung epithelium results in defective lung branching. The fusion of all four right lung lobes was observed in *Isl1*^*ShhCre*^ embryos. The results showed that the proliferation of lung mesenchymal cells was significantly reduced in *Isl1*^*ShhCre*^ embryos, while apoptosis was not altered. Coordinated epithelial–mesenchymal interactions have been shown to be critical for lung branching morphogenesis (31). Significant downregulation of mesenchymal cell proliferation suggests a link between ISL1 and secretory signaling molecules between the epithelium and mesenchyme. It should be a secreted signaling molecule that mediates the effects of *Isl1* on the mesenchymal cells.

*Sox9* and *Irx* genes are all reported to play an important role in lung branching morphogenesis (15, 16). Our results show that *Sox9*, *Irx1*, and *Irx2* are all downregulated in the *Isl1*^*ShhCre*^ embryos, suggesting that deletion of *Isl1* results in changes in the expression of multiple genes. Reciprocal communication between the epithelial layer and surrounded mesenchyme drives branching morphogenesis. During lung branching morphogenesis, many signals drive the proliferative expansion of the distal endoderm and underlying mesenchyme. Epithelial *Shh* is required for the formation of the lung lobation, branching, and growth (13). *Shh* overexpression leads to increased proliferation of mesenchymal cells (11). Therefore, we hypothesized that *Shh* acts downstream of *Isl1* to mediate the interaction between the epithelium and the mesenchyme. Previous works have shown that ISL1 regulates the expression of Shh in the tongue and urethral epithelium (32, 33). Both RNA-Seq and *in situ* hybridization results showed that *Shh* expression in the epithelium was reduced after *Isl1* deletion. The Chip analysis confirmed that ISL1 could bind to the MACS1 enhancer sequence, which strictly specifies *Shh* expression in respiratory epithelial cells (34). The involvement of Shh signaling was confirmed by rescue experiments that activate Hedgehog signaling *in vivo* and *in vitro*. The proliferation of mesenchymal cells was rescued after activating Hedgehog signaling. The number of lung branching ends significantly increased in *Isl1*^*ShhCre*^; Tg-*pmes*-*Ihh*. Moreover, upregulation of *Sox9*, *Irx1*, *Irx2*, *Tbx2*, and *Tbx3* expressions was detected in *Isl1*^*ShhCre*^; Tg-*pmes*-*Ihh*. All these data suggest that *Isl1* affects branching morphogenesis by regulating *Shh* expression.

Decreased expression of *Ptch1* confirmed a reduction in the activity of Hedgehog signaling in the lung mesenchyme of *Isl1*^*ShhCre*^. *Tbx2* and *Tbx3* express in mesenchymal cells excluding the airway epithelium during lung development and have been identified as downstream target genes of *Shh* (14). Our results indicated that both *Tbx2* and *Tbx3* expression decreased in the *Isl1*^*ShhCre*^ embryos. *Tbx2* and *Tbx3* function to maintain lung mesenchymal proliferation by regulating the expression of Cdkn1a (14, 35). The RNA-Seq data showed that cell-cycle inhibitor *Cdkn1a* was up-regulated in *Isl1*^*ShhCre*^ embryos. Downregulation of *Tbx2* and *Tbx3* might be responsible for the defective proliferation of mesenchymal cells. All these data suggest that decreased Hedgehog signaling activity in the lung mesenchyme leads to downregulation of *Tbx2* and *Tbx3*, resulting in reduced mesenchymal cell proliferation.

In summary, our data indicate transcriptional factor *Isl1* is important for lung branching morphogenesis. *Shh* expression is regulated by *Isl1* in the lung epithelium. The hedgehog signaling mediates the effect of *Isl1* between epithelium and mesenchyme. Activation of Hedgehog signaling rescues lung branching morphogenesis defects due to *Isl1* ablation. These data suggest that *Isl1* affects lung branching morphogenesis by regulating the Shh signaling pathway.

## Experimental procedures

### Animals

All of the animal experimental protocols were approved by Hangzhou Normal University Animal Care and Use Committee (2018036). Constructions of the *Isl1*^*fl*^ and *Isl1*^*LacZ*^ have been previously described (28). The Tg-*pmes*-*Ihh* mouse line was created by inserting full-length Ihh cDNA into a conditional transgenic expression vector as described previously (36). Mouse strains for *R26R-LacZ* and *Shh*^*tm1(EGFP/cre)*^ were purchased from the Jackson Laboratory and maintained on a C57BL/6J background. The morning of observed vaginal plug was designated as day 0 (E0) of pregnancy.

### X-gal staining

Whole-mount X-gal staining was performed according to the standard protocols (36). Embryos were fixed in fixing solution (4% PFA, 5 mM EGTA, and 2 mM MgCl2 in PBS) for 1 h at 4 °C. The fixed embryos were rinsed three times in washing buffer (0.02% NP40, 0.01% sodium deoxycholate, and 2 mM MgCl2 in PBS). The embryos were then incubated in staining solution (5 mM potassium ferricyanide, 5 mM potassium ferrocyanide, 2 mM Tris (pH 7.3), and 0.1% X-gal in washing buffer) 2 to 4 h in the dark at 37 °C. Finally, the stained embryos were washed in PBS and post-fixed in 4% PFA. The X-gal stained sections were counterstained with Nuclear Fast Red.

### Detection of cell proliferation and apoptosis

Cell proliferation activity was evaluated by 5-bromodeoxyuridine (BrdU) labeling and immunofluorescence staining (37). Briefly, timed pregnant mice were injected intraperitoneally with BrdU solution (3 mg/100 g of body weight) from a BrdU labeling and detection kit (Roche) 30 min before embryo harvesting. The collected embryos were fixed in 4% paraformaldehyde solution at 4 °C for 1 h and embedded in paraffin. Then embryos were processed for paraffin sectioning at 5 μm for immunohistochemical staining. BrdU-labeled cells were detected immunohistochemically in paraffin sections according to the manufacturer’s instructions. Apoptosis was assayed by TUNEL staining using the *In Situ* Cell Death Detection Kit (Roche) according to the manufacturer’s protocol. The cell proliferation rate was counted (n = 3–6 samples) and calculated as the percentage of BrdU-labeled cells among total nuclear stained cells (4′,6-diamidino-2-phenylindole [DAPI] positive) within a defined arbitrary area. The proliferation rate of control (*Shh*^*Cre*^) embryos was set to 1, and then the relative proliferation rate of *Isl1*^*ShhCre*^ mutant embryos was calculated.

### Histology, immunofluorescence, and *in situ* hybridization

Whole-mount or section immunofluorescence staining was carried out according to the standard protocol (38). For section immunofluorescence, embryos were fixed in 4% PFA for 30 min, embedded in paraffin, and sectioned at 7 μm. After blocking with 5% BSA, samples were incubated with primary antibodies at 4 °C overnight. Secondary antibodies conjugated with Alexa Fluor 488 or 594 (1:1000; Invitrogen) were applied for 30 min in the dark. Primary antibodies used were: E-cadherin (20874-1-AP; Proteintech), BrdU (ab8152, Abcam), Sox9 (mAb 82630; Cell Signaling Technology). Images were analyzed using a microscope Leica DM4 B equipped with a digital camera.

Whole-mount and section *in situ* hybridization was performed as previously described (39). For section *in situ* hybridization, Embryos were collected at the desired developmental stages and fixed in freshly made 4% paraformaldehyde (PFA) overnight at 4 °C. For whole-mount *in situ* hybridization, samples were fixed in 4% paraformaldehyde, dehydrated into methanol, and bleached with 6% hydrogen peroxide (H2O2). Non-radioactive antisense RNA probes were generated by *in vitro* transcription using DIG RNA labeling kit (Roche). For histological analysis, sections were stained with Hematoxylin and Eosin according to standard protocols.

### RNA-seq and quantitative real-time PCR

For both RNA-seq analysis and real-time RT-PCR, lungs were carefully dissected from E11.5 wild-type (*Shh*^*Cre*^) or *Isl1*^*ShhCre*^ embryos. Total RNA was extracted as previously described (40). RNA-seq libraries were established with an Illumina TruSeq RNA sample prep kit and sequenced using Illumina HiSeq 4000 with 150-bp paired-end sequencing strategy. Expression analysis was performed using HISAT2 and DESeq2, and differentially expressed genes were determined with the cutoff fold change≥1.5 and *p*-value ≤ 0.05. All the raw data generated from RNA-seq in this work was deposited in BIGD (bigd.big.ac.cn) under the accession number CRA007552. Quantitative real-time PCR was performed in triplicate for each set of samples using a CFX96 Real-Time System (Bio-Rad) and SsoFast EvaGreen Supermix (Bio-Rad). Oligonucleotide primers were designed using PRIMER3 software: *18S* (5′-TAGAGGGACAAGTGGCGTTC, and 5′-CGCTGAGCCAGTCAGTGT), *Isl1* (5′-ATGATGGTGGTTTACAGGCTAAC, and 5′- TCGATGCTACTTCACTGCCAG), *Shh* (5′-AAAGCTGACCCCTTTAGCCTA and 5′-TGAGTTCCTTAAATCGTTCGGAG), *Ptch1* (5′-AGACTACCCGAATATCCAGCACC and 5′-CCAGTCACTGTCAAATGCATCC), *Irx1* (5′- CCTATGGTCAGTTTCAATACG and 5′- GGTCATCTTGGTGATAATGG), *Irx2* (5′- ACAGGATGGCACAGAGACC and 5′- TTACACTCTGAGCCTGATTCG), *Sox9* (5′- TCCACCTTCACTTACATGAACC and 5′- AAAAAAGATCAGCTCTGTCACC), *Tbx2* (5′- AGCTGAAGATCGACAACAACC and 5′- CCTCATACAAACGGAGAGTGG), *Tbx3* (5′- GTTACAGCCCCTATTCCATCC and 5′- CCAGCAAACTGCTGCTATCC). The relative amount of gene transcript was calculated using the 2^−ΔΔCT^ method (41) and normalized to the endogenous *18S* reference gene.

### ChIP assay

ChIP analyses from E11.5 lung tissue samples were performed using a previously described protocol (36). For the binding of ISL1 to the Shh epithelial enhancer sequence (MACS1) (34), ChIP was carried out using the antibody against ISL1 (ab20670; Abcam) or normal rabbit IgG (A7016; Beyotime). For detection of the immunoprecipitated Shh epithelial enhancer sequence, eluted DNA was used as a template for triplicate quantitative real-time PCR analyses with the following primers: MACS1 B1, 5′- ATAACCAGGAGTCCAGGTAC and 5′-TCCTGAGCAGACAGAGTTCAC; MACS1 B2, 5′- GGAGAGTCCTAAGTGATCC and 5′- ACCAGTAAAAGCTTATCAGC; MFCS1, 5′-TATGACCAGATGACTTTTCC and 5′- GCCACTAACACTAAGCAGC; SBE1, 5′- GGCTGGGAGATGAACTGACC and 5′- CCTGCTATGGAGGACATGAGG.

### *In vitro* organ culture

Lung rudiments were carefully dissected from E11.5 embryos and placed on Transwell permeable membranes of 0.4-μm pore size in PET six-well plates supplied with Dulbecco's modified Eagle medium (DMEM; Gibco) supplemented with 10% fetal bovine serum and 1% penicillin/streptomycin. Purmorphamine (0.5 μM) was added to activate canonical Hedgehog signaling. Cell proliferations were monitored after 2 days of culture in a humidified atmosphere of 5% CO2 at 37 °C. 10 μM BrdU was administered to the organ culture medium and lungs were incubated for 1 h prior to PFA fixation. At least three sections per embryo were used for quantification.

### *In vitro* primary cell culture

Embryonic lungs were carefully dissected from E11.5 mouse embryos. The heart and esophagus were carefully removed. Lungs were incubated in dissociation buffer (Collagenase 100 U/ml, DNAse 100 U/ml and Trypsin 50 U/ml) for 15 min. Dissociated cells were cultured in 48 well plates. *Isl1* overexpression vector (pCDH-Isl1) or Control vector (pCDH) was transfected with Lipofectamine3000 reagent. After culturing for 48 h in a humidified atmosphere of 5% CO2 at 37 °C, the cells were used for RNA extraction and real-time PCR analysis.

### Statistical analyses

All data are presented as means ± SD. Student’s *t* test was used to test differences between two groups of data. One-way ANOVA and Tukey’s test were used to analyze the difference between multiple groups. For relative analyses wild-type values were set to 1. *p* values of < 0.05 were regarded as statistically significant.

## Data availability

All data generated or analyzed during this study are included in this published article. Materials will be made available on reasonable request.

## Conflict of interest

The authors have declared that no competing interests exist.
